# An Experience with Copper

**Published:** 1892-06

**Authors:** L. M. Stanton

**Affiliations:** New York


### AN EXPERIENCE WITH COPPER.

L. M. STANTON, M. D., NEW YORK.

June 1st, 1891, Mrs. W. came to me for treatment, with the following symptoms:

For many years she has been troubled with trembling and unsteadiness of hands, especially marked after any nervous strain. Also with cramps in the toes, feet, and calves.

These symptoms have increased much the last few months, and in addition she has been annoyed with cramps in the hands, worse in the ball of the right thumb, interfering with writing. No further symptoms could be elicited, except that a week ago she had had her usual spring "bilious attack," but was over that now.

I prescribed Cuprum-met.$^{5m}$, three doses to be taken half an hour apart, and Sac-lac.

June 4th patient reported cramps much better.

This was the last time I saw Mrs. W., as she and her daughter left town the following morning for the summer.

A few days after this I received a letter from the daughter that much surprised me.

I quote from this letter:

"Thursday night, June 4th, in New York, she (Mrs. W.) had three terrible pains just beneath her stomach, she thought the medicine you gave her was beginning to take effect.

"June 5th, traveled all day but had no pain.

"June 6th, at 2 A. M., she was seized with severe pains. At 6 A. M. I went to her room and found her in great distress. By putting her finger down her throat and with severe retching she vomited about a pint of bile, dark green. The vomiting continued at half-hour then at quarter-hour intervals with more and more thrown off, nearly a quart at times. The pain beneath the stomach and in the sides increased in severity and the doctor came at 9 A. M. He gave some pellets and I covered her bowels with a mustard plaster. Nothing took effect until 1.30 P. M. when the Morphia caused her to half sleep, but the convulsions of pain were terrible. At 1.30 the vomiting ceased, she had vomited eleven times quarts of dark green liquid. At 8 P. M. the spasms of pain were terrible and her strength gave out. Vomiting began again, this time about two cupfuls each time, black, putrid, dreadful in odor, but without retching—convulsions of pain simply threw it out of her mouth.

" At 10 P. M. the doctor injected Morphine where the pain was most cramping. Her pulse was weak. Then she slept, but cramped even in her half sleep with pain. The last vomiting was at 2 A. M.

" June 7th, the doctor came early in the morning, but she was still uneasy in pain." Something was given to move the bowels, and " between 4 and 6 P. M. she had five very large movements, first dark and green, afterward lighter. The fifth caused straining, and in examining it I found what I thought to be a gall-stone. It was the size of a damson plum.

" Mother bids me tell you she took the medicine up to the night she was taken ill, and we both feel that it must have reached the seat of all her inexplainable trouble for the past eighteen years."

Dr. Hunt, of Newtonville, Mass., who attended Mrs. W. during the attack has kindly sent me these particulars regarding the stone :

Long diameter, 1¼ inches, short diameter, ¾ inches. It has a distinct nucleus, is made up of bile, as the test of portions of the stone shows. It was perfectly hard when passed, the patient heard the stone strike the chamber.

The cramp and trembling of hands which at first I believed to be of spinal origin were unquestionably reflex, and due to the pressure of this huge stone upon the nerves supplying the gall-bladder.

Without doubt the Copper did reach the seat of trouble.

Marked and almost immediate improvement in the reflex symptoms followed the medicine. Reaction had begun; and the reaction during the next few days was waxing stronger till finally not even this stone would resist it, and its expulsion was the inevitable result.

There has, of course, been no return of the old symptoms

